# Supplementary material for: Legionella para-effectors target chromatin and promote bacterial replication
Source: Nat Commun. 2023 Apr 14;14:2154. doi: 10.1038/s41467-023-37885-z (PMC10104843; doi:10.1038/s41467-023-37885-z)
Supplement: Supplementary file 1 — Supplementary Information [file 41467_2023_37885_MOESM1_ESM.pdf]

Supplementary Information

Legionella para-effectors target chromatin and promote bacterial replication

Daniel SCHATOR [1,2,§], Sonia MONDINO [1$], Jérémy BERTHELET[3], Cristina DI SILVESTRE[1], Mathilde BEN ASSAYA[4], Christophe RUSNIOK[1], Fernando RODRIGUES-LIMA[3], Annemarie WEHENKEL[4], Carmen BUCHRIESER [1*], Monica ROLANDO[1*]

Materials and Methods

Table S1: LphD interacting proteins identified through GFP-trap analysis that are known to be involved in epigenetic regulation
Table S2: Primers used in this study
Table S3: Antibodies and dyes used in this study

Figure S1: LphD Alignment and H3/H4 peptide accommodation.
Figure S2: LphD activity on octamers and validation of anti-H3K14ac specificity.
Figure S3: LphD secretion, subcellular localization, and activity in eukaryotic cells.
Figure S4: Anti-LphD validation, representative immunoblots for histone deacetylation and complementation assays.
Figure S5: IP controls and immunoblots showing the interaction of LphD with KAT7
Figure S6: Subcellular localisation of LphD deleted of its putative NLS and H3K18ac and H3K23ac levels in LphD transfected cells.

SUPPLEMENTARY TABLES

Table S1: LphD interacting proteins identified through GFP-trap analysis that are known to be involved in epigenetic regulation

Protein	Epigenetic complex	FDR	Fold change
KAT7	HBO1	0.06	75.70
HDAC2	NuRD/Sin3	0.08	8.44
MTA2	NuRD	0.03	5.41
SAP18	Sin3	0.005	9.46
PRKDC	DNA-PK	0.005	4.71
XRCC5	DNA-PK	0.02	14.47
SSRP1	FACT	0.02	3.08
SUPT16H	FACT	0.001	3.39
EED	PRC2/EED-EZH2	0.03	32.19
SUZ12	PRC2/EED-EZH2	0.002	15.45
EZH2	PRC2/EED-EZH2	0.09	9.17

Table S2: Primers used in this study

Primer	Sequence (5'-3')	Purpose	Reference
52H	GATGAAGGCACGAACCCAGTTGACA	Deletion of *lphD* gene	This study
52B	CGGCTTGAACGAATTGTTAGGTGGC	Deletion of *lphD* gene	This study
195H	GACCCTCGACTTAATTGGATAACGG	Deletion of *lphD* gene	This study
195B	GGAGGGTAAACGGAAAACAAACTG	Deletion of *lphD* gene	This study
196H	GCCACCTAACAATTCGTTCAAGCCGGGTGATC<br>TAAGCTACTTCTATGAATCCATATTC	Deletion of *lphD* gene	This study
196B	TGTCAACTGGGTTCGTGCCTTCATCGTTTCCTG<br>AGTTGTAATAATTGGTAAATTCC	Deletion of *lphD* gene	This study
11H	TCCAATATACAAGCATTCATGTGCTATCTG	Deletion of *romA* gene	[1]
11B	GAAGTTTCTCGAATTCTTTGGACAAGC	Deletion of *romA* gene	[1]
60H	CATCGATGAATTGTGTCTCAAAA	Deletion of *romA* gene	[1]
60B	GTCCCGTCAAGTCAGCGTA	Deletion of *romA* gene	[1]
66H	TTTTGAGACACAATTCATCGATG GCTCTATTT<br>TGCATGTGATTTTCATT	Deletion of *romA* gene	[1]
66B	TACGCTGACTTGACGGGAC GCAAGTTTTTTG<br>ATTTGATATTTCCTG	Deletion of *romA* gene	[1]
217H	ATCTATTGGGGCTTGGAAGGTGGATTTGACAG<br>GACCATGTATGAA	Base pair substitution (LphD<br>Y392F)	This study
217B	TTCATACATGGTCCTGTCAAATCCACCTTCCAA<br>GCCCCAATAGAT	Base pair substitution (LphD<br>Y392F)	This study
222H	GGATCCGAAAGTAGCGCCTCTTGTCTTGATA	Complementation of *lphD*	This study
222B	GGTACCTTAACAGGACATATTATGCGAATTGG	Complementation of *lphD*	This study
CP_07H	CTCTCAATCTCCAGCCACAAAT	CXCL1 qPCR ChIP	[1]
CP_07B	CCTGAGAACCACCACAGAGAAG	CXCL1 qPCR ChIP	[1]
CP_26H	GCTCCCCAACCTAGTGTCAT	MyD88 qPCR ChIP	[1]
CP_26B	GGAGTGGGAAACGGACAG	MyD88 qPCR ChIP	[1]
CP_37H	AGTTGTGGTCTGTGGCACTC	IFNB1 qPCR ChIP	[1]
CP_37B	AGTTTGGGCTTTCTCACAGC	IFNB1 qPCR ChIP	[1]
CP_30H	GATGTGGAGCTGGGATGTC	BCL2L1 qPCR ChIP	[1]
CP_30B	CATGGCAGCAGTAAAGCAAG	BCL2L1 qPCR ChIP	[1]
CP_22H	TGAGAAAGGAGGTGGGTAGG	IL6 qPCR ChIP	[1]
CP_22B	CCCAGCAAAGACCTCCTAAT	IL6 qPCR ChIP	[1]
AJUBA_fwd	CCAGGAATCCCACAGCATCC	AJUBA qPCR ChIP	[1]
AJUBA_rev	CGGGGAGAGTGGGGCA	AJUBA qPCR ChIP	[1]
TXNIP_fwd	ATTGGGCCGCTTACCTGTTG	TXNIP qPCR ChIP	[1]
TXNIP_rev	GTTAGGATCCTGGCTTGCGG	TXNIP qPCR ChIP	[1]

Table S3: Antibodies and dyes used in this study

Target	Manufacturer	Product code	Dilution
Western blot:			
b-Actin	Sigma	A5316	10.000
BRPF1	Active Motif	61541	1000
EGFP	Thermo Fisher	A11122	2000
FLAG	Sigma	F3165	2000
GST	Milipore	AB3282	1000
H1	Active Motif	61201	2000
H3	Active Motif	39163	2000
H3K14ac	Milipore	07-353	2000
H3K14me2	Euromedex	H3-2B10	3000
H3K18ac	Active Motif	39755	1000
H3K23ac	Active Motif	39131	1000
HA	Sigma	H6908	5000
HDAC1	Santa Cruz Biotechnology	sc-81598	500
HIS	Sigma	H1029	2000
ING5	Active Motif	91329	500
KAT7	Santa Cruz Biotechnology	sc-39846	500
MEAF6	Thermo Fisher	PA5-40704	1000
V5	Thermo Fisher	46-0705	3000
anti-Mouse HRP-conjugated	Cell signaling	7076S	2500
anti-Rabbit HRP-conjugated	Cell signaling	7074S	2500
Immunofluorescence:			
LphD	Thermo Fisher	Custom (see **Methods**)	500
Alexa488 goat anti-Rabbit	Thermo Fisher	A32731	1000
Alexa546 goat anti-Rabbit	Thermo Fisher	A11010	1000
DAPI	Thermo Fisher	D21490	600
Alexa633 phalloidin	Thermo Fisher	A22284	500

REFERENCES

1. Rolando, M. *et al. Legionella pneumophila* effector RomA uniquely modifies host chromatin to repress gene expression and promote intracellular bacterial replication. *Cell Host Microbe* 13, 395–405 (2013).

2. Jumper, J. *et al.* Highly accurate protein structure prediction with AlphaFold. *Nature* 596, 583–589 (2021).

Figure S1

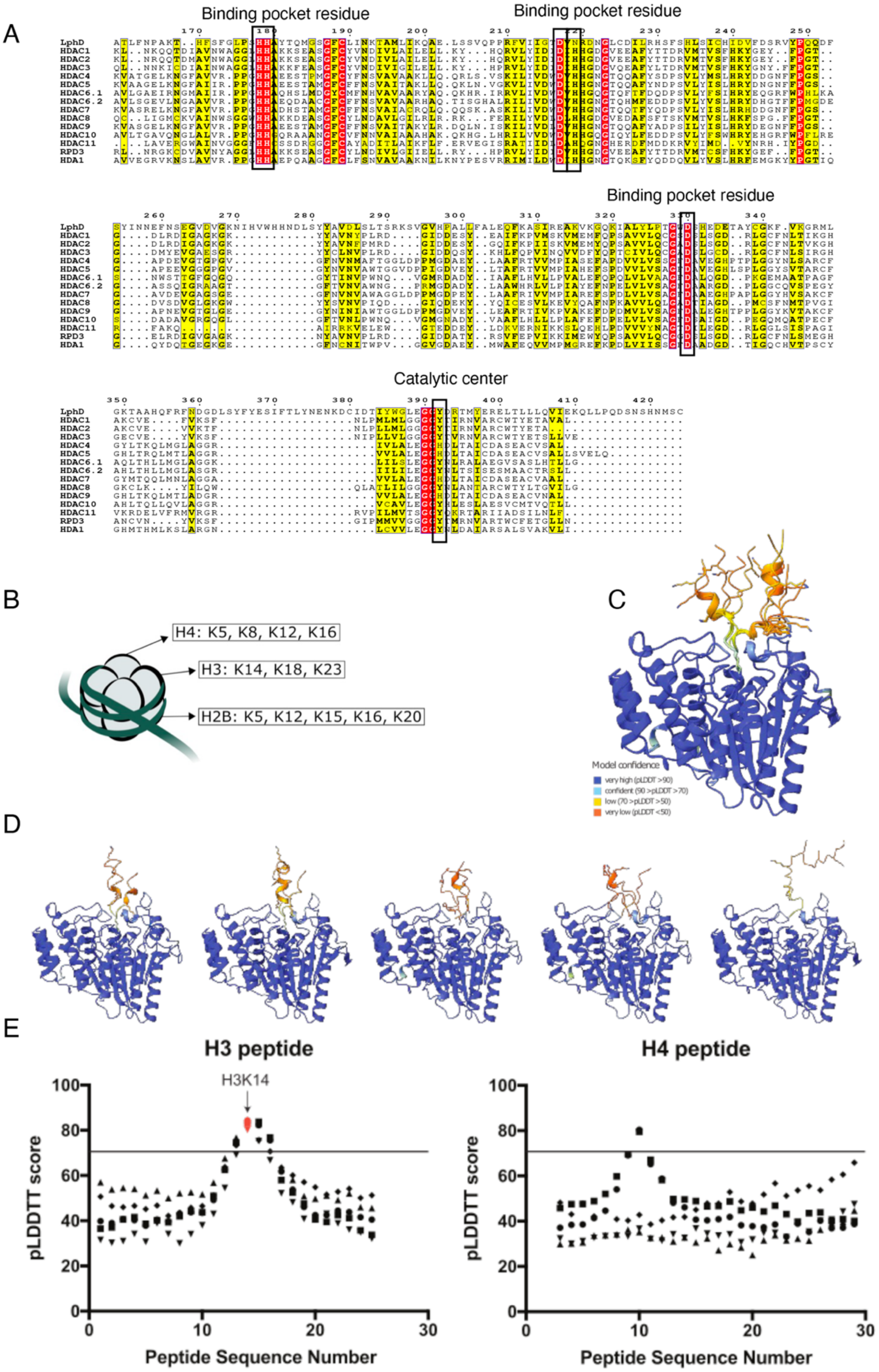

Figure S1: LphD Alignment and H3/H4 peptide accommodation. (**S1A**) Multiple sequence alignment of LphD (aa 160-424) and several eukaryotic HDAC domains (Accession numbers: *Homo sapiens*: HDAC1 #Q6IT96, HDAC2 #Q92769, HDAC3 #O15379, HDAC4 #P56524, HDAC5 #Q9UQL6, HDAC6.1 #Q9UBN7, HDAC6.2 #Q9UBN7, HDAC7 #Q8WUI4, HDAC8 #Q9BY41,

HDAC9 #Q9UKV0, HDAC10 #Q969S8, HDAC11 #Q96DB2; *Saccharomyces cerevisiae*: Rpd3 # P32561, HDA1 #P53973). Analysis also includes two yeast HDACs (Rpd3 and HDA1), which are the basis of general HDAC classification. Alignment was performed using SeaView and visualization with ESPript. Red boxes with white characters mean strict identity between all samples, bold characters mean high in-group similarity and yellow boxes mean high across-group similarity. Binding pocket residues (charge relay system) and the catalytic center are marked with black frames. (**S1B**) Histone tail residues identified as possible targets of LphD by MS/MS. Purified histones were incubated with or without purified LphD for 1 hour at 37ºC, followed by MS/MS analysis. (**S1C**) Cartoon representation of the LphD-histone tail (H3) model generated by AlphaFold[2]. The per-residue confidence score (pLDDT) produced by AlphaFold is shown in the insert. Superposition of the 5 models of the LphD-H3 peptide complex. All 5 models place the same lysine 14 into the active site pocket. (**S1D**) Cartoon representation of the LphD-histone tail models (H4) generated by AlphaFold. Individual models of LphD-H4 complexes showing different binding modes for the H4 peptide. Only the first two place a lysine residue into the active site. (**S1E**) Graphical representation of pLDDT scores (y axis) for H3 peptide (left) and H4 peptide (right) predictions. Each symbol corresponds to a model generated by AF. The 70% cutoff line corresponding to a confident prediction is shown. The H3K14 is highlighted in red and marked by an arrow.

Figure S2

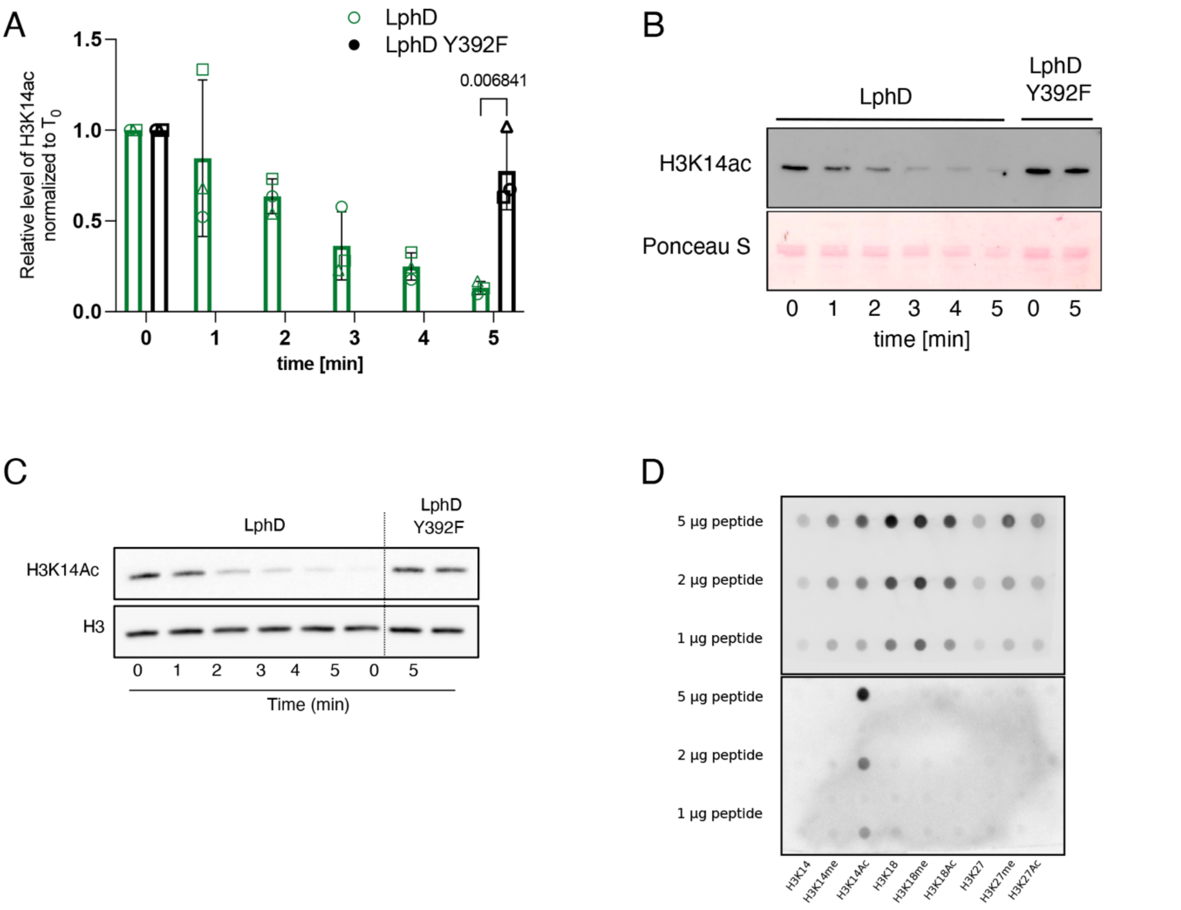

Figure S2: LphD activity on octamers and validation of anti-H3K14ac specificity. (**S2A**) Densiometry quantification of LphD activity on H3K14ac levels on high-acetylated histone octamers. Octamers are incubated with LphD (green) and LphD Y392F (black) and the reaction was stopped after the indicated time (in minutes). The H3K14ac signal was quantified after immunoblot detection and normalized to the signal at 0 min (n = 3 ± SD). (**S2B**) Representative western blot of LphD activity on H3K14ac levels on high-acetylated histone octamers. Wild type protein (LphD) is compared to the catalytic inactive mutant (LphD Y392F) and H3K14ac levels are assessed every minute for 5 minutes by immunoblot. Ponceau S staining of H3 is used as loading control. (**S2C**) Representative western blot of LphD activity on H3K14ac levels on nucleosomes. Wild type protein (LphD) is compared to the catalytic inactive mutant (LphD Y392F). H3K14ac levels are assessed every minute for 5 minutes by immunoblot. H3 is used as loading control. (**S2D**) Validation of H3K14ac antibody specificity by dot blot experiments, using different amounts of several H3 peptides (H3K14, H3K14me, H3K14ac, H3K18, H3K18me, H3K18ac, H3K27, H3K27me, H3K27ac).

Figure S3

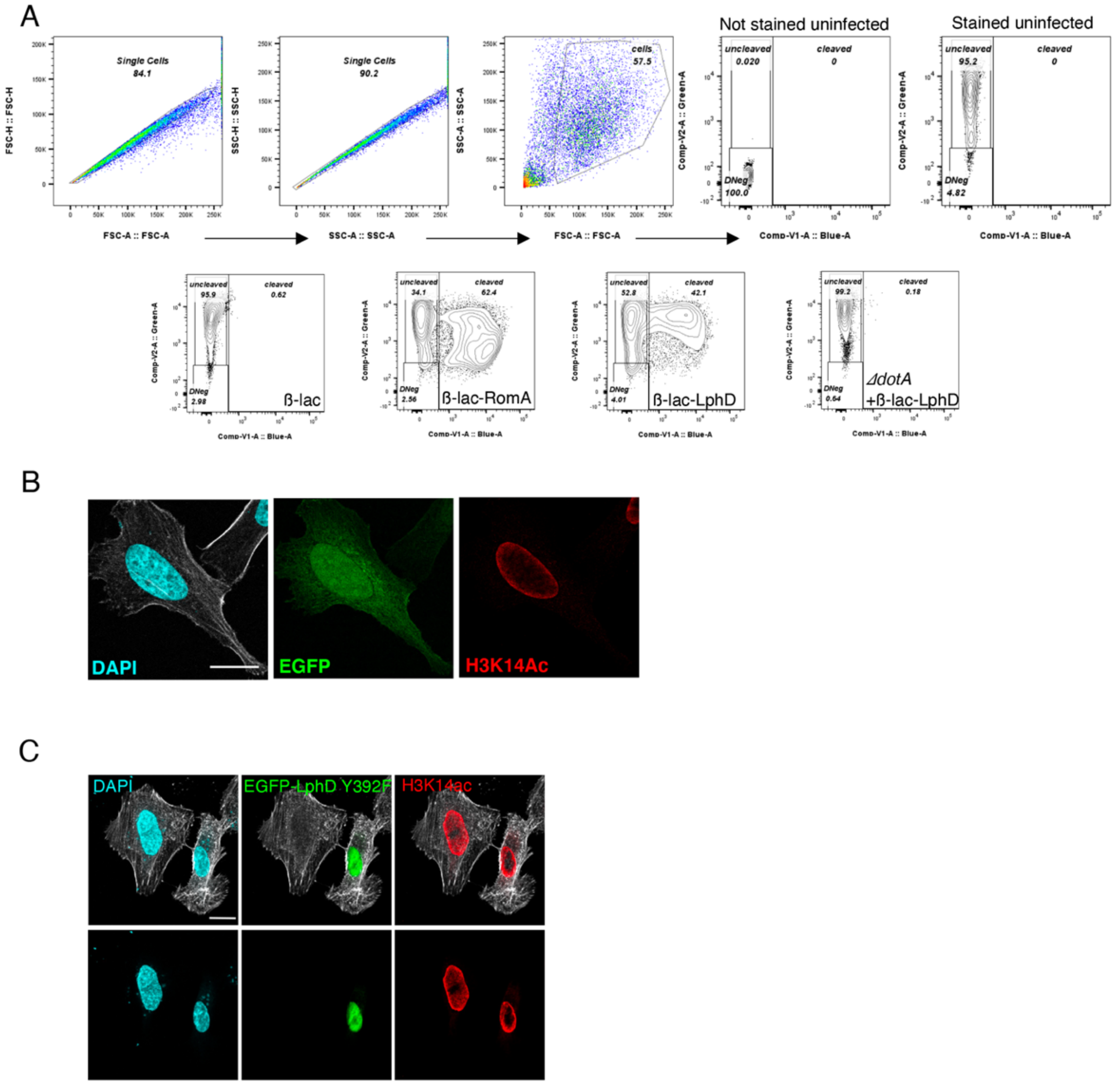

Figure S3: LphD secretion, subcellular localization, and activity in eukaryotic cells. (**S3A**) FACS Strategy of ß-lactamase secretion assay. Representative image of using FSC vs SSC plot to gate on live cells. THP1 cells were infected, as indicted, and after CCF4 loading, samples were analyzed by flow cytometry to determine green (Y axis) and blue (X axis) cell populations, where green represents no translocation and blue represents translocation of the ß-lactamase into the cytosol. Double negative (DNeg; uninfected unstained) cells were used as negative control. Stained uninfected cells were used to gate the green positive cells (uncleaved CCF4). Representative gates of the analyzed conditions: ß-lac (uncleaved CCF4); ß-lac-RomA (positive control: cleaved CCF4 inducing a cell shift in blue channel); ß-LphD (as for the positive control: cleaved CCF4 and blue shift); ß-LphD in a Δ*dotA* strain (uncleaved CCF4). (**S3B**) Immunofluorescence analysis of subcellular localization of EGFP. HeLa cells were transfected with EGFP. DAPI (cyan), EGFP (green), and phalloidin (gray). Scale bars 10 µm. (**S3C**) Immunofluorescence analysis of subcellular localization of EGFP-LphD Y392F and H3K14 acetylation. HeLa cells were transfected with EGFP-LphD Y392F for 24hours and then stained for H3K14ac using a specific antibody. DAPI (cyan), EGFP-LphD Y392F (green), H3K14ac (red), and phalloidin (gray). Single channel images are shown. Scale bars 10 µm. (**D**) Immunofluorescence analysis of subcellular localization of LphD during infection. Differentiated THP-1 cells were infected 16 hours at an MOI of 50 with *L. pneumophila* wild type expressing V5-LphD and GFP. DAPI (cyan), V5-LphD (yellow), *L. pneumophila* (green), and H3K14ac (red). In the first panel uninfected (NI) and infected (I) cells are framed and zoomed in panels NI and I, respectively. Scale bars 10 µm.

Figure S4

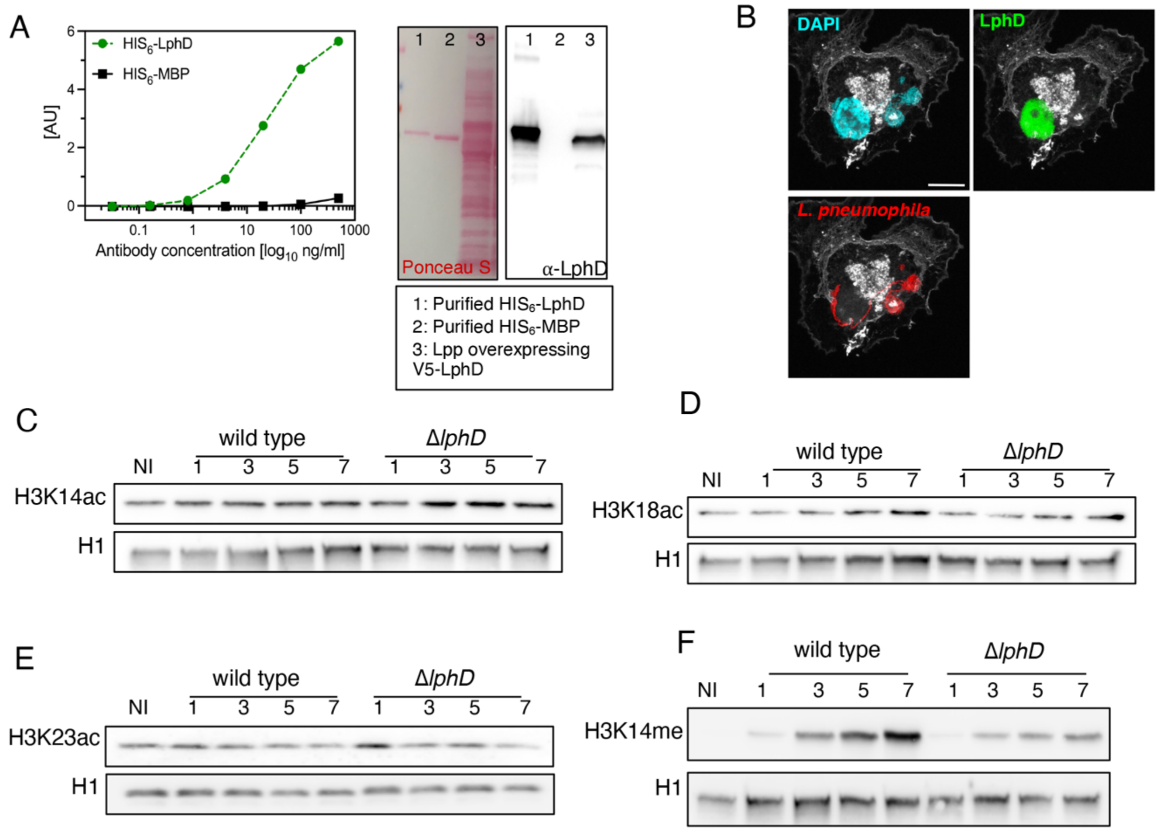

Figure S4: Anti-LphD validation, representative immunoblots for histone deacetylation and complementation assays. (**S4A**) Validation of custom anti-LphD antibody produced in rabbit. **Left**: Titer determination of polyclonal antibodies by ELISA comparing binding activity to purified HIS_6-LphD, compared to HIS_6-MBP. **Right**: Specificity testing by western blot using anti-LphD antibody. Line 1: purified HIS_6-LphD, Line 2: purified HIS_6-MBP, Line 3: *L. pneumophila* extract overexpressing V5-LphD. (**S4B**) Immunofluorescence analysis of subcellular localization of LphD during infection. Differentiated THP-1 cells were infected 16 hours at an MOI of 50 with *L. pneumophila* wild type expressing LphD and DsRed. DAPI (cyan), LphD (green), *L. pneumophila* (red), and phalloidin (gray). Scale bars 10 µm. (**S4C, D, E, F**) Representative image of western blots for H3K14ac (**S4C)**, H3K18ac (**S4D**), H3K23ac (**S4E**), and H3K14me (**S4F**). THP-1 cells were infected with *L. pneumophila* wild type or Δ*lphD* strain expressing EGFP. Cells were sorted at different times post-infection by FACS, histones were isolated and analyzed by western blot. Histone H1 was used as loading control.

Figure S5

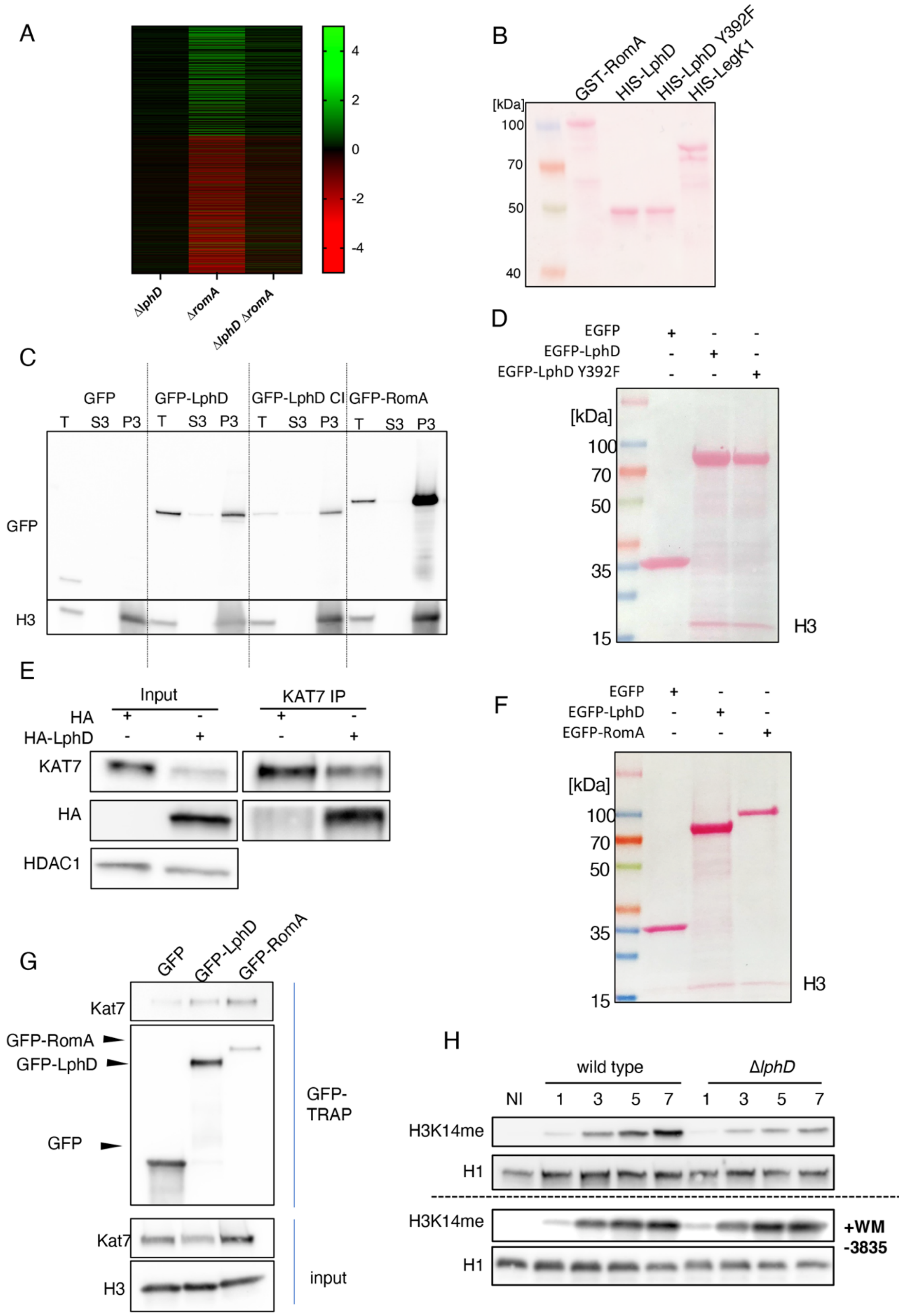

Figure S5: IP controls and immunoblots showing the interaction of LphD with KAT7. (**S5A**) Heat map of RNAseq results. Genes significantly up- (log_2 fold change ≥ 2, p.adjust < 0.1) or down-regulated (log_2 fold change ≥ -2, p.adjust < 0.1) (~4000 genes) during wild type *L. pneumophila* infection are analyzed and the impact of the three knockout strains (Δ*lphD*, Δ*romA* and Δ*lphD*Δ*romA*) on these genes during infection is shown in terms of log_2 fold change. THP-1 cells were infected with *L. pneumophila* wild type, Δ*lphD*, Δ*romA* or Δ*lphD*Δ*romA* strains expressing EGFP. Cells were sorted 7 hours post-

infection by FACS and RNA processed for the RNAseq (n = 3). (**S5B**) Input of **Figure 4A**. Ponceau S staining of tagged forms of proteins used for *in vitro* binding assay, as indicated. (**S5C**) LphD and RomA are chromatin associated. Biochemical fractionation (see Material and Methods for details) of HEK293T cells transfected with EGFP, EGFP-LphD, EGFP-LphD Y392F or EGFP-RomA for 24 or 48 hours. LphD (wild type and CI) as well as RomA are enriched in the chromatin enriched fraction (P3). H3 was used as loading control for the chromatin fraction (T: total; S3: nuclear soluble; P3: nuclear insoluble/chromatin associated). Experiments were repeated twice. (**S5D**) IP control of **Figure 4E**. Ponceau S staining of GFP-trap samples showing the corresponding immunoprecipitated products (EGFP = 27 kDa, EGFP-LphD/ EGFP-LphD Y392F = 75 kDa). (**S5E**) Immunoblots showing the interaction of LphD with KAT7. Co-immunoprecipitation of endogenous KAT7 in HEK293T cells transfected with 3xHA or 3xHA-LphD. Input shows the expression level of endogenous KAT7 and HA-LphD in total lysates, as well as HDAC1 (loading control). Co-IP samples were analyzed for the presence of 3xHA-LphD. (**S5F**) IP control of Figure 4F. Ponceau S staining of GFP-trap samples showing the corresponding immunoprecipitated products (EGFP = 27 kDa, EGFP-LphD = 75 kDa and EGFP-RomA=88kDa). (**S5G**) DNA-independent association between KAT7 and LphD or RomA. GFP-trap beads (GFP-trap) in HEK293T cells transfected with EGFP, EGFP-LphD or EGFP-LphD RomA. The IP were done in presence of Ethidium Bromine (400µg/ml). Input shows the expression level of endogenous KAT7 and Histone H3 in total lysates. IP panel shows immunoprecipitated endogenous KAT7 as well as GFP signal. Experiments were repeated twice. (**S5H**) Representative image of western blots for H3K14me, corresponding to. THP-1 cells were pre-treated 18 hours with a KAT7 specific inhibitor (WM-3835) and infected with *L. pneumophila* wild type or Δ*lphD* strain expressing EGFP. Cells were sorted at different times post-infection by FACS, histones were isolated and analyzed by western blot. Histone H1 was used as loading control.

Figure S6

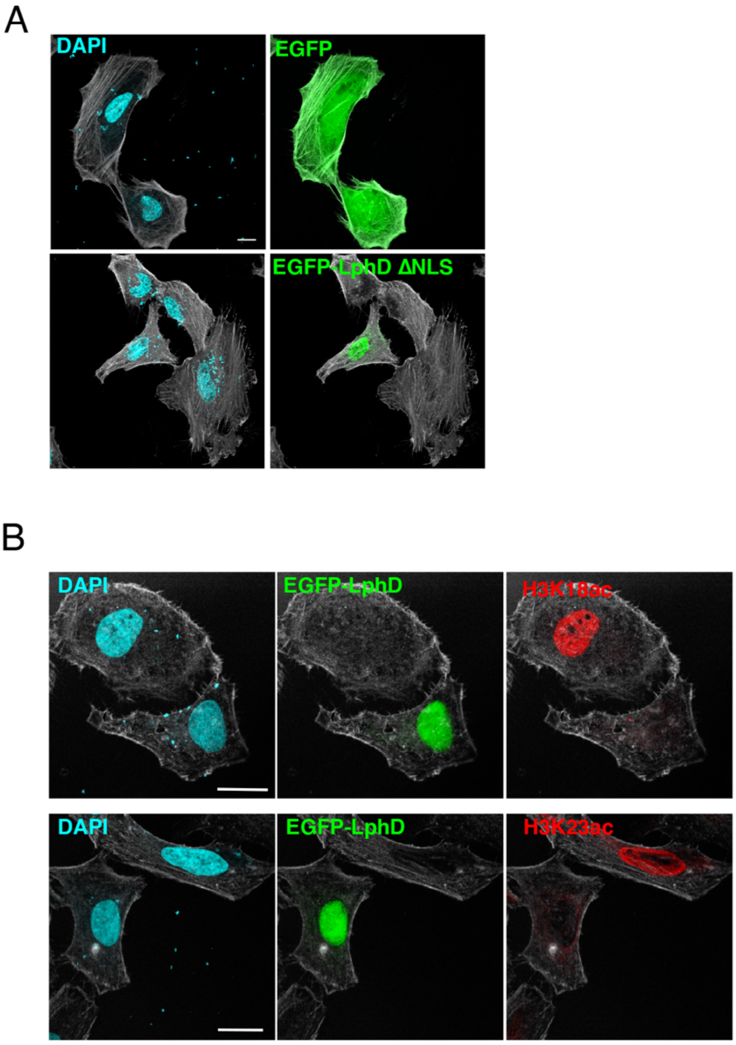

Figure S6: Subcellular localisation of LphD deleted of its putative NLS and H3K18ac and H3K23ac levels in LphD transfected cells. (**S6A**) Immunofluorescence analysis of subcellular localization of EGFP-LphD full-length and a truncated form for its predicted NLS corresponding to amino acids 2-22 in the N-terminal part of the protein (prediction tool NoD: NucleOlar localization sequence Detector[3]). HeLa cells were either transfected with EGFP-LphD or with EGFP-LphD ΔNLS for 24 hours and then stained with DAPI (cyan), and phalloidin (gray). Scale bars 10 µm. (**S6B)** Immunofluorescence analysis of H3K14ac levels of cells transfected with EGFP-LphD. HeLa cells were transfected 24 hours with EGFP-LphD and stained for H3K18Ac (red; top) or H3K23Ac (red; bottom), DAPI (cyan), and phalloidin (gray). Scale bars 10 µm.
