# Supplementary material for: Legionella para-effectors target chromatin and promote bacterial replication
Source: Nat Commun. 2023 Apr 14;14:2154. doi: 10.1038/s41467-023-37885-z (PMC10104843; doi:10.1038/s41467-023-37885-z)
Supplement: Supplementary file 3 — Reporting Summary [file 41467_2023_37885_MOESM3_ESM.pdf]

nature portfolio

Corresponding author(s): Buchrieser Carmen, Monica Rolando

Last updated by author(s): Mar 15, 2023

# Reporting Summary

Nature Portfolio wishes to improve the reproducibility of the work that we publish. This form provides structure for consistency and transparency in reporting. For further information on Nature Portfolio policies, see our Editorial Policies and the Editorial Policy Checklist.

## Statistics

For all statistical analyses, confirm that the following items are present in the figure legend, table legend, main text, or Methods section.

| n/a | Confirmed | |
|---|---|---|
| ☐ | ☒ | The exact sample size ($n$) for each experimental group/condition, given as a discrete number and unit of measurement |
| ☐ | ☒ | A statement on whether measurements were taken from distinct samples or whether the same sample was measured repeatedly |
| ☐ | ☒ | The statistical test(s) used AND whether they are one- or two-sided<br>*Only common tests should be described solely by name; describe more complex techniques in the Methods section.* |
| ☐ | ☒ | A description of all covariates tested |
| ☐ | ☒ | A description of any assumptions or corrections, such as tests of normality and adjustment for multiple comparisons |
| ☐ | ☒ | A full description of the statistical parameters including central tendency (e.g. means) or other basic estimates (e.g. regression coefficient) AND variation (e.g. standard deviation) or associated estimates of uncertainty (e.g. confidence intervals) |
| ☐ | ☒ | For null hypothesis testing, the test statistic (e.g. $F$, $t$, $r$) with confidence intervals, effect sizes, degrees of freedom and $P$ value noted<br>*Give P values as exact values whenever suitable.* |
| ☒ | ☐ | For Bayesian analysis, information on the choice of priors and Markov chain Monte Carlo settings |
| ☒ | ☐ | For hierarchical and complex designs, identification of the appropriate level for tests and full reporting of outcomes |
| ☒ | ☐ | Estimates of effect sizes (e.g. Cohen's $d$, Pearson's $r$), indicating how they were calculated |

*Our web collection on statistics for biologists contains articles on many of the points above.*

## Software and code

Policy information about availability of computer code

| | |
|---|---|
| Data collection | no software was used for data collection |
| Data analysis | Data analysis is described in the Methods section. Graph were plotted using GraphPad Prism 9 v9.5.0; Statistical analyses was done in GraphPad Prism 9 v9.5.0; Image analysis was performed using Fiji/ImageJ v2.9.0/1.53t and MultiGauge v3.0.; Structure predictions were performed using AlphaFold v2.0.1; Flow cytometry data was analyzed using FlowJo v10.8; multiple sequence alignemants performed using using SeaView v5.0.4 and visualization with web-based ESPript v3.0; qPCR data analyzed in QuantStudio Thermo Fisher Scientific v2.6.0; Michaelis–Menten equation fitted curves using OriginPro v8.0. MGF peak files from Progenesis were processed by Proteome Discoverer v2.4 with the Sequest search engine. Sequence reads were analyzed with the STAR alignment (v2.5.2b) – DESeq2 (v1.14.1) software pipeline |

For manuscripts utilizing custom algorithms or software that are central to the research but not yet described in published literature, software must be made available to editors and reviewers. We strongly encourage code deposition in a community repository (e.g. GitHub). See the Nature Portfolio guidelines for submitting code & software for further information.

## Data

Policy information about availability of data

All manuscripts must include a data availability statement. This statement should provide the following information, where applicable:

- Accession codes, unique identifiers, or web links for publicly available datasets
- A description of any restrictions on data availability
- For clinical datasets or third party data, please ensure that the statement adheres to our policy

The main data supporting the findings of this study are available within the article and its Supplementary Figures. The source data underlying figures and Supplementary Figures are provided as Source Data File.
To use Proteome Discoverer a custom database was created using the Swissprot/TrEMBL protein database release 2019_08 with the Homo sapiens taxonomy and including LphD, both from the Legionella pneumophila taxonomy.
The sequence reads as well as the coverage files of the RNAseq libraries of THP-1 cells have been deposited in the NCBI Gene Expression Omnibus (GEO) database 48. Accession number GSE207487.

## Human research participants

Policy information about studies involving human research participants and Sex and Gender in Research.

| | |
|---|---|
| Reporting on sex and gender | na |
| Population characteristics | na |
| Recruitment | na |
| Ethics oversight | na |

Note that full information on the approval of the study protocol must also be provided in the manuscript.

# Field-specific reporting

Please select the one below that is the best fit for your research. If you are not sure, read the appropriate sections before making your selection.

☒ Life sciences ☐ Behavioural & social sciences ☐ Ecological, evolutionary & environmental sciences

For a reference copy of the document with all sections, see nature.com/documents/nr-reporting-summary-flat.pdf

# Life sciences study design

All studies must disclose on these points even when the disclosure is negative.

| | |
|---|---|
| Sample size | In general, no calculations were done to determine sample size. Sample size was determined based on standards for experimental cell biology and animal studies, attempting to have a minimum of N = 3 biological replicates with sufficient reproducibility. Figs. 1C, 1F, 2A, 3D, 3E, S2A includes technical replicates per biological replicates. |
| Data exclusions | No data have been excluded |
| Replication | Experiments were replicated as stated in the method sections and figure legends. |
| Randomization | None of the experimental methods used in this study necessitated any randomization of sample groups. |
| Blinding | Immunofluorescence analyses and cfu counting were performed blinded. The rest of the analysis are considered as objective measures, not subject to bias and therefore the integrity of the results are not impacted when running the study and analysis unblinded. |

# Reporting for specific materials, systems and methods

We require information from authors about some types of materials, experimental systems and methods used in many studies. Here, indicate whether each material, system or method listed is relevant to your study. If you are not sure if a list item applies to your research, read the appropriate section before selecting a response.

## Materials & experimental systems

| n/a | Involved in the study | |
|---|---|---|
| ☐ | ☒ | Antibodies |
| ☐ | ☒ | Eukaryotic cell lines |
| ☒ | ☐ | Palaeontology and archaeology |
| ☒ | ☐ | Animals and other organisms |
| ☒ | ☐ | Clinical data |
| ☒ | ☐ | Dual use research of concern |

## Methods

| n/a | Involved in the study | |
|---|---|---|
| ☒ | ☐ | ChIP-seq |
| ☐ | ☒ | Flow cytometry |
| ☒ | ☐ | MRI-based neuroimaging |

# Antibodies

Antibodies used

b-Actin: Sigma A5316 1:10.000
BRPF1: Active Motif 61541 1:1000
GFP: Thermo Fisher A11122 1:2000
FLAG: Sigma F3165 1:2000
GST: Milipore AB3282 1:1000
H1: Active Motif 61201 1:2000
H3: Active Motif 39163 1:2000 (5 µl for ChIP)
H3K14ac: Milipore 07-353 1:2000
H3K14ac: Abcam ab52946 1:10000
H3K14me: Euromedex H3-2B10 1:3000 (5 µl for ChIP)
H3K18ac: Active Motif 39755 1:1000
H3K23ac: Active Motif 39131 1:1000
HA: Sigma H6908 1:5000
HDAC1: Santa Cruz Biotechnology sc-81598 1:500
HIS: Sigma H1029 1:2000
ING5: Active Motif 91329 1:500
KAT7: Santa Cruz Biotechnology sc-398346 1:500
MEAF6: Thermo Fisher PA5-40704 1:1000
V5: Thermo Fisher 46-0705 1:3000
anti-Mouse HRP-conjugated: Cell signaling 7076S 1:2500
anti-Rabbit HRP-conjugated: Cell signaling 7074S 1:2500
Custom LphD: Thermo Fisher 1:500 (5 µl for ChIP)
Alexa488 goat anti-Rabbit: Thermo Fisher A32731 1:1000
Alexa546 goat anti-Rabbit: Thermo Fisher A11010 1:1000
Alexa633 phalloidin: Thermo Fisher A22284 1:500
IgG: Abcam ab46540 (2 µl for ChIP)

Validation

H3K14ac antibody was validated for specificity in FigS2D by dot blot experiments, using different amounts of several H3 peptides (H3K14, H3K14me, H3K14ac, H3K18, H3K18me, H3K18ac, H3K27, H3K27me, H3K27ac).
LphD antibody specificity was validated by Western blot of purified LphD, as well as bacterial extracts (overexpressing LphD). In addition, ELISA was performed to assess antibody sensitivity (Fig S4A)

Please find here the technical data sheets of the manufacturers the other antibodies:
b-Actin: https://www.sigmaaldrich.com/certificates/Graphics/COfAInfo/sigmail01/pdf/A5316_128K4805.pdf
BRPF1: https://www.activemotif.com/documents/tds/61541.pdf
GFP: hhttps://www.thermofisher.com/order/genome-database/dataSheetPdf?producttype=antibody&productsubtype=antibody_primary&productId=A-11122&version=225
FLAG: https://www.sigmaaldrich.com/certificates/COFA/F1/F1804/F1804-BULK________SLCD3524__.pdf
GST: https://www.merckmillipore.com/FR/en/product/Anti-Glutathione-S-Transferase-Antibody-S.-japonicum-form,MM_NF-AB3282#anchor_COA (LOT: LV1691082)
H1: https://www.activemotif.com/documents/tds/61201.pdf
H3: https://www.activemotif.com/documents/tds/39163.pdf
H3K14ac: https://www.merckmillipore.com/FR/en/product/Anti-acetyl-Histone-H3-Lys14-Antibody,MM_NF-07-353#anchor_COA (LOT: 3011823A)
H3K14ac:
H3K14me: https://shopresearch.euromedex.com/document/FTPDF/IG-H3-2B10.pdf
H3K18ac: https://www.activemotif.com/documents/tds/39755.pdf
H3K23ac: https://www.activemotif.com/documents/tds/39131.pdf
HA: https://www.sigmaaldrich.com/certificates/COFA/H6/H6908/H6908-BULK________099M4865V_.pdf
HDAC1: https://datasheets.scbt.com/sc-81598.pdf
HIS: https://www.sigmaaldrich.com/certificates/Graphics/COfAInfo/sigmail01/pdf/H1029_097M4894V.pdf
ING5: https://www.activemotif.com/documents/tds/91329.pdf
KAT7: https://datasheets.scbt.com/sc-398346.pdf
MEAF6: https://www.thermofisher.com/order/genome-database/dataSheetPdf?producttype=antibody&productsubtype=antibody_primary&productId=PA5-40704&version=225
V5: https://www.thermofisher.com/order/genome-database/dataSheetPdf?producttype=antibody&productsubtype=antibody_primary&productId=R960-25&version=225
anti-Mouse HRP-conjugated: https://www.cellsignal.com/datasheet.jsp?productId=7076&images=1&size=A4
anti-Rabbit HRP-conjugated: https://www.cellsignal.com/datasheet.jsp?productId=7074&images=1&size=A4

Alexa488 goat anti-Rabbit: https://www.thermofisher.com/order/genome-database/dataSheetPdf?producttype=antibody&productsubtype=antibody_secondary&productId=A32731&version=225
Alexa546 goat anti-Rabbit: https://www.thermofisher.com/order/genome-database/dataSheetPdf?producttype=antibody&productsubtype=antibody_secondary&productId=A-11010&version=225
IgG: https://www.abcam.com/rabbit-mouse-igg-hl-ab46540.html

## Eukaryotic cell lines

Policy information about cell lines and Sex and Gender in Research

| | |
|---|---|
| Cell line source(s) | HeLa from ATCC (CCL2), THP-1 from ATCC (TIB-202), HEK 293T from ATCC (CRL-11268), HEK 293 FcGamma RII from the lab of Craig Roy - published https://doi.org/10.1111/j.1600-0854.2010.01050.x |
| Authentication | Standard cell lines were used; no particular authentification process was conducted |
| Mycoplasma contamination | All cell lines were tested for Mycoplasma contamination and tested negative. |
| Commonly misidentified lines (See ICLAC register) | We did not use any commonly misidentified cell lines. |

## Flow Cytometry

### Plots

Confirm that:

☒ The axis labels state the marker and fluorochrome used (e.g. CD4-FITC).

☒ The axis scales are clearly visible. Include numbers along axes only for bottom left plot of group (a 'group' is an analysis of identical markers).

☒ All plots are contour plots with outliers or pseudocolor plots.

☒ A numerical value for number of cells or percentage (with statistics) is provided.

### Methodology

| | |
|---|---|
| Sample preparation | THP1 cells were detached by addition of cell dissociation solution and analyzed in suspension in the flow cytometer |
| Instrument | BIORAD S3 cell sorter / MACS Quant, Miltenyi Biotec |
| Software | FlowJo™ v10.8 |
| Cell population abundance | Fig S3: we present around 20,000 unsorted infected cells per condition. |
| Gating strategy | Total cell population gated on single cells then on size and structure of live cells (excluding debris). Then gating green and blue to detect stained cells (green channel) and cleaved-CCF4 positive cells (blue channel). Gating details in Fig S3A |

☒ Tick this box to confirm that a figure exemplifying the gating strategy is provided in the Supplementary Information.
